# Transcriptional changes during isoproterenol-induced cardiac fibrosis in mice

**DOI:** 10.3389/fmolb.2023.1263913

**Published:** 2023-12-18

**Authors:** Disha Nanda, Priyanka Pant, Pratheusa Machha, Divya Tej Sowpati, Regalla Kumarswamy

**Affiliations:** ^1^ Council of Scientific and Industrial Research (CSIR)–Centre for Cellular and Molecular Biology, Hyderabad, India; ^2^ Academy of Scientific and Innovative Research (AcSIR), Ghaziabad, India

**Keywords:** cardiac fibrosis, ECM remodeling, lncRNAs, isoproterenol, transcriptomics

## Abstract

**Introduction:** β-adrenergic stimulation using β-agonists such as isoproterenol has been routinely used to induce cardiac fibrosis in experimental animal models. Although transcriptome changes in surgical models of cardiac fibrosis such as transverse aortic constriction (TAC) and coronary artery ligation (CAL) are well-studied, transcriptional changes during isoproterenol-induced cardiac fibrosis are not well-explored.

**Methods:** Cardiac fibrosis was induced in male C57BL6 mice by administration of isoproterenol for 4, 8, or 11 days at 50 mg/kg/day dose. Temporal changes in gene expression were studied by RNA sequencing.

**Results and discussion:** We observed a significant alteration in the transcriptome profile across the different experimental groups compared to the saline group. Isoproterenol treatment caused upregulation of genes associated with ECM organization, cell–cell contact, three-dimensional structure, and cell growth, while genes associated with fatty acid oxidation, sarcoplasmic reticulum calcium ion transport, and cardiac muscle contraction are downregulated. A number of known long non-coding RNAs (lncRNAs) and putative novel lncRNAs exhibited differential regulation. In conclusion, our study shows that isoproterenol administration leads to the dysregulation of genes relevant to ECM deposition and cardiac contraction, and serves as an excellent alternate model to the surgical models of heart failure.

## Introduction

Cardiovascular diseases are a major cause of mortality, and their prevalence continues to increase (Lindstrom et al., 2022). Cardiac injury causes myocardial remodeling, which often results in heart failure (Azevedo et al., 2015). The end-stage heart failure is often accompanied by systolic dysfunction and increased interstitial fibrosis (Almehmadi et al., 2014).

Surgical models of heart failure, including transverse aortic constriction (TAC) and coronary artery ligation (CAL), are valuable tools used in cardiovascular research to study the mechanisms and progression of heart failure, and to evaluate potential therapeutic interventions. TAC induces left ventricular pressure overload by constricting the transverse aorta and replicates the chronic progressive nature of heart failure development as seen in patients (Richards et al., 2019). CAL involves the surgical occlusion of coronary arteries, resulting in myocardial ischemia and subsequent heart failure. It replicates aspects of heart failure caused by coronary artery disease (Gao et al., 2010). Although these models are invaluable in cardiovascular research, they require surgical expertise, involve a high mortality rate, and may induce systemic effects beyond the heart, such as inflammatory responses and changes in neurohormonal signaling.

Hyperactivity of the sympathetic nervous system is the central feature of heart failure. This brings about an increase in the level of catecholamines such as norepinephrine, which stimulate β-adrenergic receptors creating a compensatory adaptive response that transiently normalizes the cardiac output by thickening the ventricular wall (Triposkiadis et al., 2009). However, prolonged stimulation of β-adrenergic receptors sustains the process of cardiac remodeling with an increase in the ventricular lumen and cardiac fibrosis. β-adrenergic stimulation using β-agonists such as isoproterenol has been routinely used to induce cardiac diseases experimentally in animal models. A low dose of isoproterenol 10 mg/kg/day (Grant et al., 2020) induces cardiac hypertrophy whereas a higher dose of isoproterenol (100 mg/kg/day) causes cardiomyocyte necrosis, followed by fibrosis (Mitra et al., 2013). Compared to the surgical models, using β-agonist to induce heart failure is quick and less arduous. There are several studies discerning the transcriptional changes post-MI (Ounzain et al., 2015) or TAC (Matkovich et al., 2014). However, global transcriptome changes during β-agonist-induced myocardial remodeling are not well-studied. In this study, we explored temporal transcriptional changes during isoproterenol-induced cardiac fibrosis in mice. Our data show that genes related to ECM remodeling, cardiac contraction, and several noncoding RNAs are deregulated as early as 4 days after isoproterenol treatment.

## Methods

### Animal work

Animal experiments were performed according to local and national ethics guidelines. Experimental mice were maintained on an *ad libitum* diet with free access to water under a standard 12-h light/12-h dark cycle. Here, 12-week-old C57BL6/J male mice were subjected to a daily subcutaneous dose of isoproterenol 50 mg/kg/day (Tocris #1747) for a period of 4, 8, and 11 days; as a control, few mice were given saline doses for a similar period. For the experiments, each group had three or more mice. Mice were euthanized by cervical dislocation, and hearts were perfused with 30 mM KCl to induce diastolic arrest, followed by perfusion with PBS (phosphate-buffered saline). A cross section of each heart was collected in 4% paraformaldehyde (PFA) for histology, and a small amount was snap-frozen for RNA isolation.

### Histology

A formaldehyde-fixed cross section of the heart was dehydrated by passing them through a series of increasing concentrations of isopropanol and was embedded in paraffin. Paraffin-embedded tissues were sectioned at 4 μm and placed on the charged slides. Deparaffinization was conducted by dipping slides in xylene and subsequently passing them through the series of reducing isopropanol gradients.

For wheat germ agglutinin (WGA) staining, Alexa Fluor™ 488 conjugate (#W11261, Invitrogen) was diluted at 1:100 dilution from a stock solution of 1 mg/mL and placed over each section. Sections were kept in the dark for 40 mins, followed by PBS wash and counterstained with DAPI 1 μg/mL dissolved in PBS. Slides were washed thrice with PBS, and the coverslip was mounted using the ProLong Gold Antifade medium. Images were taken with an Olympus FV3000 confocal microscope at ×60. ImageJ was used for measuring the area of individual cardiomyocytes.

For picrosirius staining, the picrosirius red stain (Abcam, ab150681) was applied onto the deparaffinized sections and kept for incubation at RT for 60 mins. The slides were rinsed in two changes of acetic acid solution, followed by dehydration in two changes of absolute alcohol. Coverslips were mounted using DPX. ImageJ was used to analyze collagen deposition in these sections.

### Cardiac cell fractionation

Adult mouse cardiomyocytes were isolated using the Langendorff-free method, where the aorta was clamped and the heart is continuously perfused through the left ventricle with the help of a syringe (Ackers-Johnson et al., 2016). The protocol was modified slightly. Briefly, the adult mouse was anesthetized with ketamine (100 mg/kg) and xylaxine (10 mg/kg). To prevent coagulation of blood, the animals were injected with 0.1 mL of heparin (100 I.U./mouse). Chest cavity of the anesthetized mouse was opened to expose the heart and inferior vena cava.

The descending aorta was cut, and the right ventricle was perfused with prewarmed 10 mL perfusion buffer (113 mM NaCl, 4.7 mM KCl, 0.6 mM KH_2_PO_4_, 0.6 mM Na_2_HPO_4_, 1.2 mM MgSO_4_-7H_2_O, 0.032 mM phenol red, 12 mM NaHCO_3_, 10 mM KHCO_3_, 10 mM HEPES, 30 mM taurine, 0.1% glucose, and 10 mM 2,3-butanedione monoxime). Ascending aorta was located by gently pulling the LV apex toward the diaphragm. The aorta was clamped with the help of curved hemostatic forceps, and the tissue behind the clamp was cut to excise the heart. The clamped heart was transferred to a 60-mm dish containing 10 mL of perfusion buffer. A measure of 5 mL of perfusion buffer was injected into the apex of the left ventricle to remove all the blood. The heart was transferred to a new 60-mm dish having 10 mL of digestion buffer (perfusion buffer supplemented with 700 U/mL collagenase II, Worthington, #LS004177, and 12.5 µM CaCl_2_). Prewarmed digestion buffer was injected into the apex of the left ventricle, and the heart was slowly and continuously perfused. Perfusion was continued till the tissue became flaccid. Atria were removed, and ventricles were shifted to a new dish; the tissue was teased apart gently with the help of forceps. A measure of 1 mL of collagenase buffer was added, and the tissue was triturated with a 1-mL pipette tip with a wide opening. The supernatant was collected and passed through a 100-μm cell strainer into a 50-mL centrifuge tube having 2 mL FBS (fetal bovine serum) for inactivation of collagenase. This cell suspension was transferred to a 15-mL centrifuge tube, and it was kept for gravity settling at 37°C for 20 mins. Rod-shaped cardiomyocytes being heavy sediments and supernatant enriched in non-myocyte cells were collected in a new tube. The supernatant was further subjected to centrifugation at 0.1 g for 1 min to aid the settling of round cardiomyocytes. All of the non-myocyte cell suspension was pooled by centrifugation at 0.8 g for 5 min, and 1 mL TRIzol was added to both myocyte and non-myocyte cell pellets. RNA was isolated as described.

### RNA isolation and qRT-PCR

The snap-frozen tissue section was pulverized in TRIzol (RNAiso Plus, TaKaRa) using the Precellys homogenizer. RNA isolation was performed according to the manufacturer’s instructions. The resulting RNA pellet was resuspended in nuclease-free water. To remove any remaining genomic DNA, the RNA samples were treated with DNase I (NEB #M0303). A portion of the DNA-free RNA samples was then used for cDNA synthesis using the PrimeScript 1st Strand cDNA Synthesis Kit (TaKaRa #6110A), following the manufacturer’s instructions. The SYBR Green Master Mix (TaKaRa #RR820) was used for quantitative RT-PCR using primers given in Supplementary Table S1.

### RNA sequencing

Total RNA was extracted from the saline, 4-, 8-, or 11-day isoproterenol-treated hearts in replicates of four. Sequencing libraries were prepared using the Illumina TruSeq total RNA kit. To ensure data quality, we performed FastQC for raw read quality control (Andrews, 2010). Raw reads were then filtered and processed using Cutadapt v4.1 (Martin, 2011). This involved trimming adapter sequences, removing low-quality bases with a quality cutoff of 20, and discarding reads below a minimum length of 50. The trimmed reads were aligned to the GRCm38.p6 mouse reference genome using the HISAT2 v2.2.1 aligner (Kim et al., 2015) with default parameters. To identify and eliminate outliers in the dataset, principal component analysis (PCA) was performed on the rlog normalized counts. One replicate from the 8-day drug-treated condition was excluded from further analysis due to quality issues.

### Pipeline for prediction of novel lncRNAs

To consolidate RNA-seq alignments into transcripts, we employed StringTie’s v2.1.7 (Pertea et al., 2015) merge mode to generate a consistent set of transcripts for all samples. For expression estimation and transcript-level expression profiling, StringTie was then utilized in conjunction with GENCODE v25 (https://www.gencodegenes.org/mouse/) as the reference annotation file. Subsequently, a set of inclusion criteria was applied to the transcripts obtained from the output GTF file (Ounzain et al., 2015; Azlan et al., 2019; Ward et al., 2021). The criteria were as follows: (i) the transcripts should be multi-exonic; (ii) they should correspond to gffcompare v0.11.2 (Pertea and Pertea, 2020) class codes ‘u' (intergenic), ‘i' (intronic), ‘x' (antisense), or ‘y' (containing a reference gene within its intron) based on the reference annotation GENCODE v25 (Ward et al., 2021); (iii) the transcript length should exceed 200 nucleotides; (iv) the transcripts should align to the autosomes or the X chromosome; (v) they should lack coding potential and possess an open reading frame (ORF) shorter than 300, as assessed by the Coding Potential Assessment Tool (CPAT v3.0) (Clark et al., 2015; Chen et al., 2016) using default settings,; (vi) transcripts identified as coding regions by TransDecoder v5.5 (https://github.com/TransDecoder/TransDecoder) and those with homology to the UniProt dataset determined by BLASTP were discarded; (vii) transcripts with significant hits for known protein domains, identified using HMMER against the Pfam database (Finn et al., 2014), were also discarded; and (viii) transcripts with a read count less than 5 across all samples were excluded.

### Differential gene expression analysis

To conduct pairwise differential expression analysis, we employed DeSeq2 v1.36 (Love et al., 2014), a widely used R package for studying differential gene expression. Our analysis involved comparing the saline group with each of the three drug-treated time-point experimental groups individually. This comprehensive approach allowed us to identify significant variations in the gene expression at different time points. We imported both the gene and transcript level counts into DeSeq2 using the tximport v1.24 (Soneson et al., 2015) package. Transcripts with a sum of raw counts less than 10 across all samples were excluded from the analysis. The remaining filtered transcripts were then normalized using DeSeq2’s “geometric” normalization strategy. Genes showing a log fold change (LFC) greater than or equal to an absolute value of 0 and an adjusted *p*-value cutoff of *p* < 0.05 were considered differentially regulated genes (DEGs). For the putatively novel lncRNAs identified, we determined their statistically significant differential expression within each specific time experimental group. We considered transcripts with an absolute log fold change (LFC) greater than 1 and a significance threshold of alpha = 0.01 to be indicative of differential expression.

### Putative novel lncRNA–mRNA correlation test

We utilized the abundance values obtained from tximport for all the differentially expressed putative novel lncRNA transcripts and protein-coding genes (PEGs) exhibiting a LFC value greater than an absolute value of 1. These abundance values were then subjected to an association analysis using Pearson’s correlation test. To account for multiple comparisons, we applied the Benjamini–Hochberg correction. Statistically significant associations between novel lncRNA and protein-coding gene (DEL:DEG) pairs were identified based on a padj value less than 0.05.

### Time-course analysis

In order to identify genes exhibiting significant expression changes throughout the different timepoints of drug treatment, we employed maSigPro v1.68 (Conesa et al., 2006), a regression-based approach that detects clusters of genes with similar temporal expression profiles. Gene abundances were quantified using RSEM v1.3.3 (Li and Dewey, 2011), and fragments per kilobase of transcript per million read (FPKM) counts were obtained. Genes with a count less than 10 across all samples were excluded from the analysis. A regression model with a degree of 3 was constructed, and hits with a false discovery rate (FDR) of 0.05 (Q = 0.05) were considered significant.

### Statistics

Data analyses were conducted using GraphPad Prism 9.0. One way ANOVA, followed by Dunnett’s test/Fishers test for multiple comparisons, was performed to compute statistical differences. Quantitative data are presented as means ± SD, and statistical significance was achieved when *p* ≤ 0.05.

## Results

### Isoproterenol treatment leads to changes in the morphology of the heart

**FIGURE 1:**
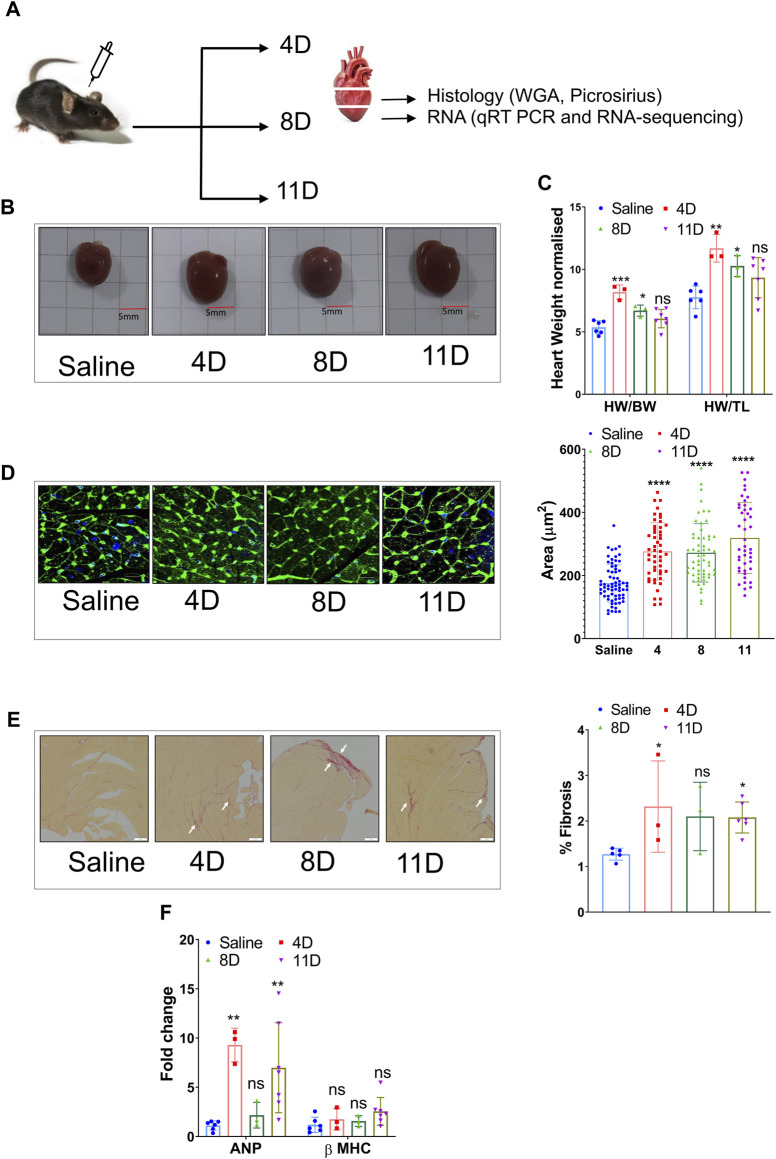
Induction of cardiac hypertrophy with a subcutaneous dose of isoproterenol. **(A)** Overview of the experimental setup where 12-week-old C57BL6 male mice were administered with 50 mg/kg/day isoproterenol for 4, 8, and 11 days, and the heart samples were collected for histology and RNA isolation. **(B)** Representative images of the hearts that were collected at different time points after isoproterenol treatment. **(C)** Graph showing heart weights normalized to body weight (BW) and tibia length (TL) at definitive timepoints. **(D)** Representative WGA staining and cardiomyocyte size analysis. **(E)** Picrosirius red staining indicative of ECM remodeling among the three groups and its quantification (n>=3) (marked in white arrow). **(F)** qRT PCR showing the levels of heart failure markers like ANP and β-MHC. * *p* value < 0.05, ** *p* value < 0.01, *** *p* value < 0.001, and **** *p* value < 0.0001. One way ANOVA followed by Dunnett’s test/Fishers test was performed to compute statistical differences between saline and isoproterenol groups.

In this study, adult 12-week-old male mice were given a subcutaneous dose of isoproterenol at 50 mg/kg/day for 4, 8, and 11 days (Figure 1A), which led to a significant increase in heart weight and size when normalized to both body weight and tibia length (Figures 1B, C). The heart samples were collected at respective time points and sectioned for real-time PCR (qRT PCR) and histology (Figure 1A). There was an increase in the cross-sectional area of cardiomyocytes compared to the saline group (Figure 1D). Additionally, the isoproterenol-treated groups exhibited collagen deposition, as evident from picrosirius red staining (Figure 1E). qRT-PCR analysis further revealed elevated expression of cardiac stress markers such as *Anp* (atrial natriuretic peptide) and *Myh7* (myosin heavy chain 7) (Figure 1F).

### Changes in the cardiac transcriptome during isoproterenol-induced fibrosis

**FIGURE 2:**
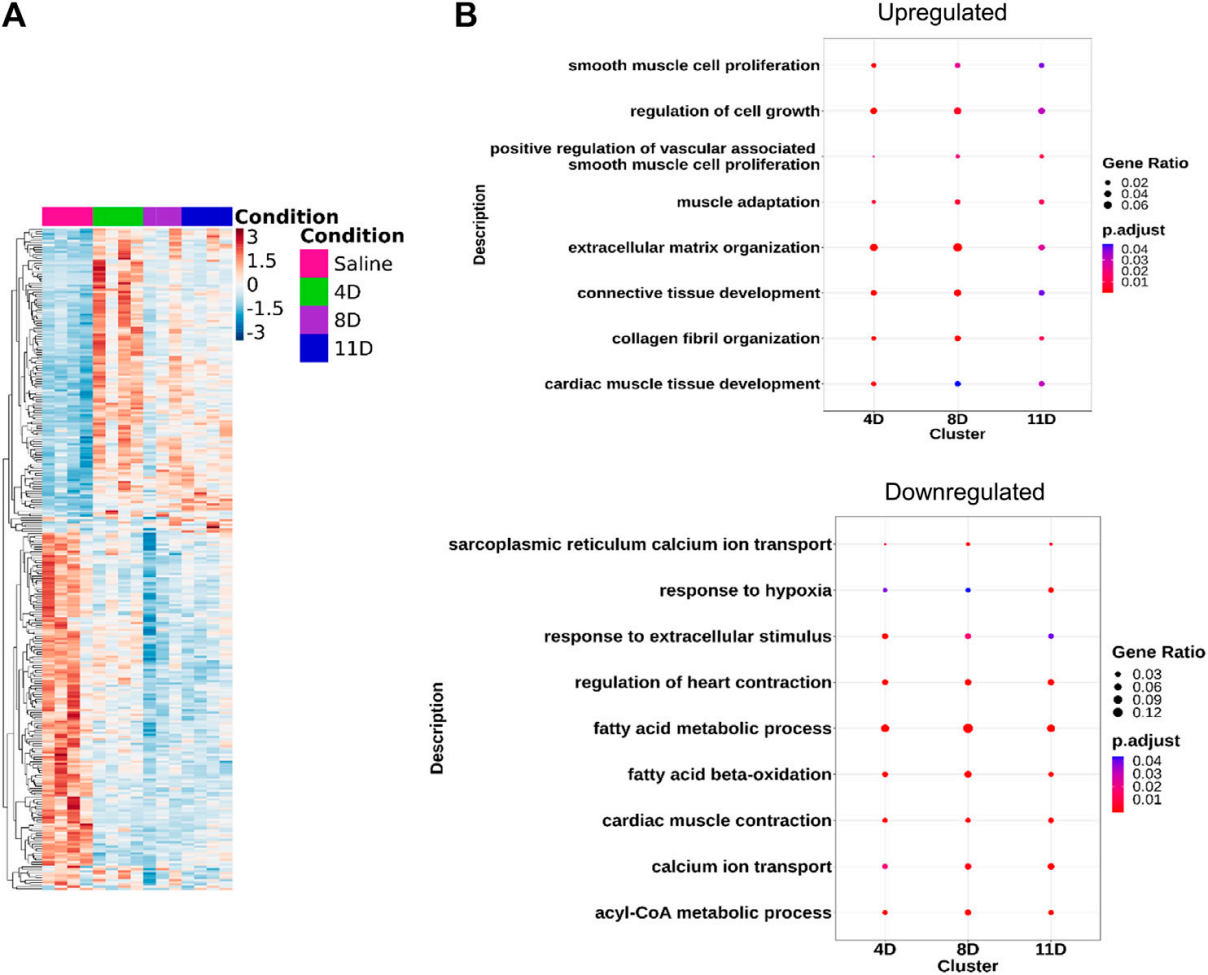
Transcriptomic analysis of the isoproterenol-treated hearts. RNA sequencing shows differential expression of common genes (DEGs) across the three groups. **(A)** Heatmap showing the DEGs across the three timepoints compared to control (LFC = 0, *p* < 0.05). **(B)** Gene Ontology showing the common pathways affected during isoproterenol-induced cardiac remodeling.

Ventricular RNA samples were obtained from mice that received subcutaneous injections of isoproterenol and saline. These samples were then subjected to RNA-seq analysis, resulting in an average of 56.4 ± 31.5 million uniquely mapped reads per sample. The analysis revealed approximately 296 common genes that exhibited significant differences compared to the saline group, with an FDR-adjusted *p*-value < 0.05 (Figure 2A). In the 4-day isoproterenol treatment group, a total of 1548 genes were upregulated and 915 genes were downregulated (Supplementary Figure S1A; Supplementary Table S2). In the 8-day group, 372 genes were upregulated and 425 genes were downregulated (Supplementary Figure S1A and Supplementary Table S2), while the 11-day treatment group had 500 upregulated genes and 591 downregulated genes (Supplementary Figure S1A; Supplementary Table S2). Gene Ontology analysis identified enrichment of ECM organization, maintaining cell–cell contact, three-dimensional structure, and cell growth regulatory genes in the upregulated gene set, indicating augmented cardiac remodeling and fibrosis (Figure 2B, Supplementary Table S3). Re-expression of several fetal genes like *Acta1* and *Pdlim1* was also observed, which is a hall mark of pathogenic remodeling (Supplementary Table S2). Downregulated genes primarily carried out fatty acid oxidation, sarcoplasmic reticulum calcium ion transport, and cardiac muscle contraction, indicating attenuated cardiac contractility (Figure 2B; Supplementary Table S3). Other signaling pathways that play a very important role in cardiac physiology like ERK, WNT, and calcium signaling were also disrupted (Supplementary Table S3).

**FIGURE 3:**
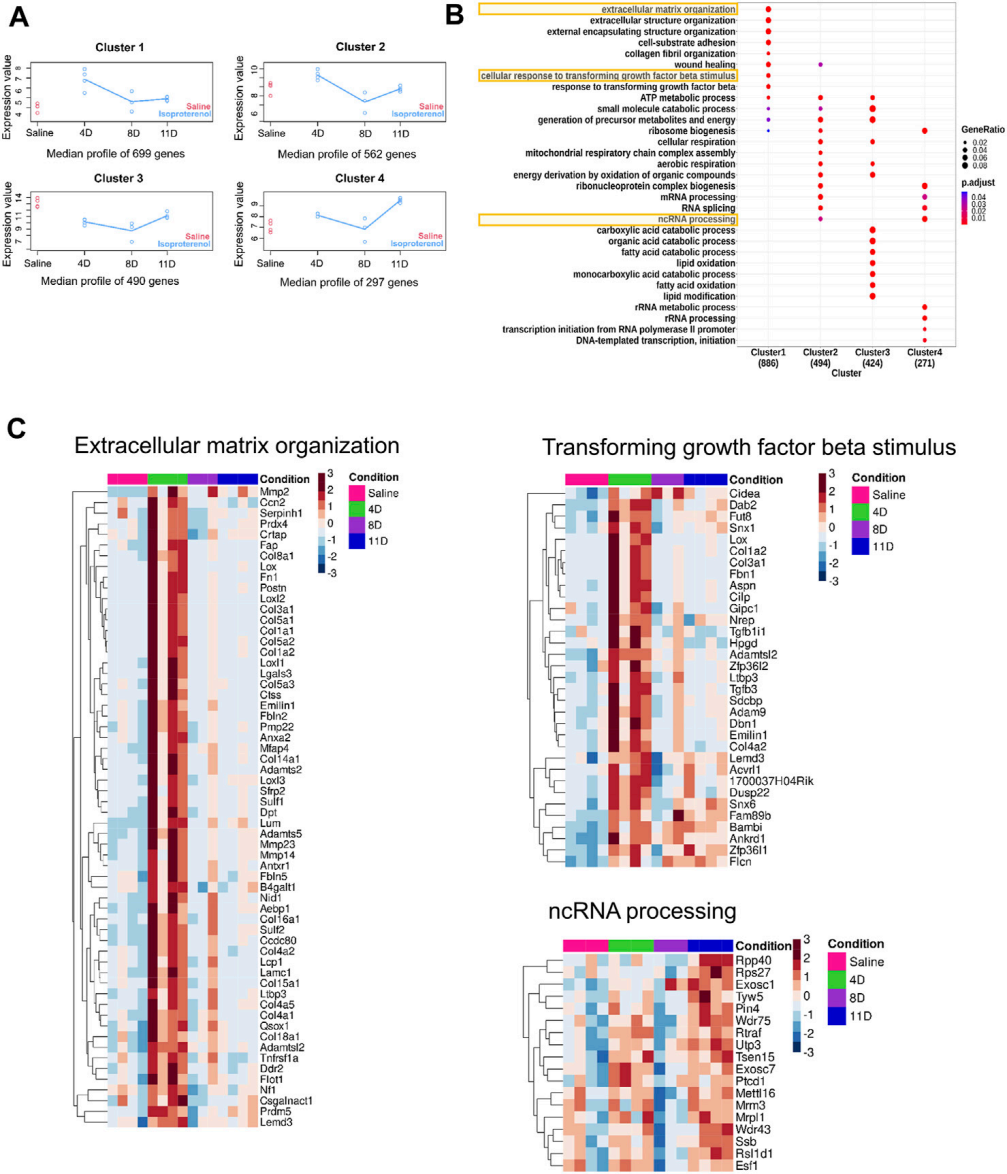
Time-course analysis of DEGS **(A)** Time-course analysis shows significant changes in gene expression over time. The gene expression profile was grouped into four clusters with distinct temporal profiles. **(B)** Gene Ontology (GO) of the genes obtained from the aforementioned four clusters. **(C)** Heatmap depicting the gene expression patterns of few pathways from each clusters.

To analyze genes with significantly different expression profiles over time during isoproterenol-induced fibrosis, we utilized the maSigPro tool, which grouped them into four clusters depending on the biological processes they regulate (Figure 3A). Genes in clusters 1 and 2 exhibited an increased expression at 4 days upon isoproterenol treatment with slow reduction in expression over time (Figures 3A, B; Supplementary Tables S4, S2). Genes in cluster 1 primarily function in extracellular matrix maintenance (Figures 3A, B; Supplementary Tables S4, S2). These include different collagens (*Col1*, *Col3*, and *Col15*), structural proteins (*Adamts* and *Mmps*), and profibrotic markers (*Postn*, *Tgfbr2*, *Fbln7*, and *Fn1*) (Figures 3B, C). Genes in cluster 2 regulate some important processes like respiration and ribosome biogenesis, and are severely hampered upon sustained cardiac stress (Supplementary Figure S2). Cluster 3 primarily included genes related to fatty acid and lipid metabolism, and genes in this cluster are downregulated upon isoproterenol treatment (Figure 3B; Supplementary Figure S2; Supplementary Tables S4, S5). Cluster 4 mainly consisted genes regulating RNA processing and splicing, and these genes are upregulated during isoproterenol-induced fibrosis at 11 days (Figure 3B; Supplementary Figure S2; Supplementary Tables S4, S5). Similar to the temporal expression changes, we also observed increased collagen deposition in the respective animal groups from as early as 4 days after isoproterenol injection (Figure 1E).

### Altered long non-coding RNA expression profile during isoproterenol-induced cardiac fibrosis

Previous studies have revealed changes in the expression of lncRNAs, following transverse aortic constriction (TAC) or myocardial infarction (MI). In this investigation, we present novel findings on the differential expression of lncRNAs upon isoproterenol treatment for 4, 8, or 11 days (Supplementary Table S6). Notably, well-characterized lncRNAs such as *Snhg4* and *Lockd* were deregulated upon isoproterenol treatment (Supplementary Table S6).

**FIGURE 4:**
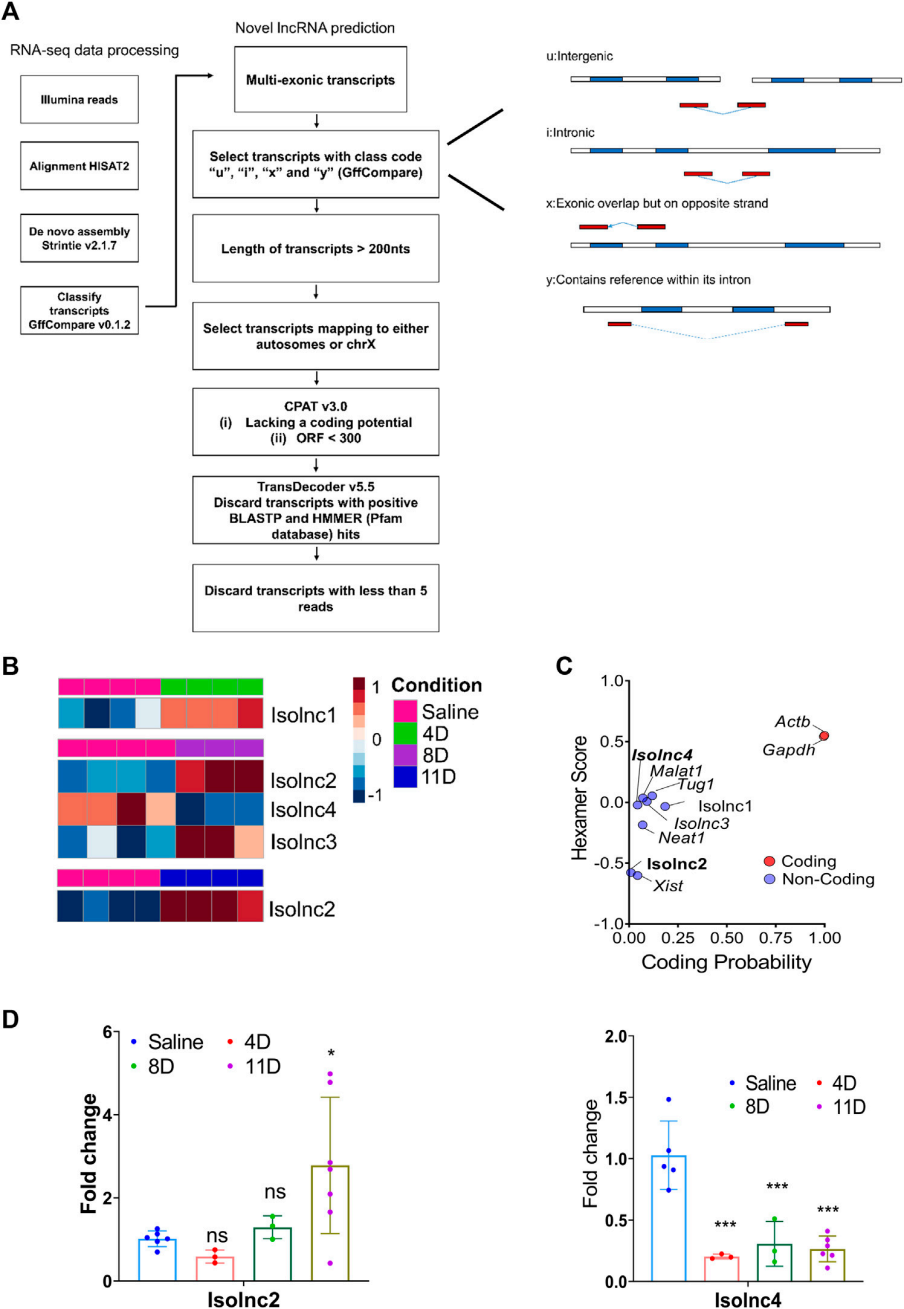
Expression profile of novel long non-coding RNA transcripts. **(A)** Schematic representation of the novel lncRNA prediction pipeline. **(B)** RNA sequencing shows differential expression of different novel lncRNAs across three groups. **(C)** Analysis of coding potential of Isolncs by CPAT compared to known coding and non-coding RNAs. **(D)** qRT PCR validation of Isolncs 2 and 4 in isoproterenol-treated animals. * *p* value < 0.05, ** *p* value < 0.01, and *** *p* value < 0.001. One-way ANOVA followed by Dunnett’s test was performed to compute statistical differences between saline and isoproterenol groups.

**FIGURE 5:**
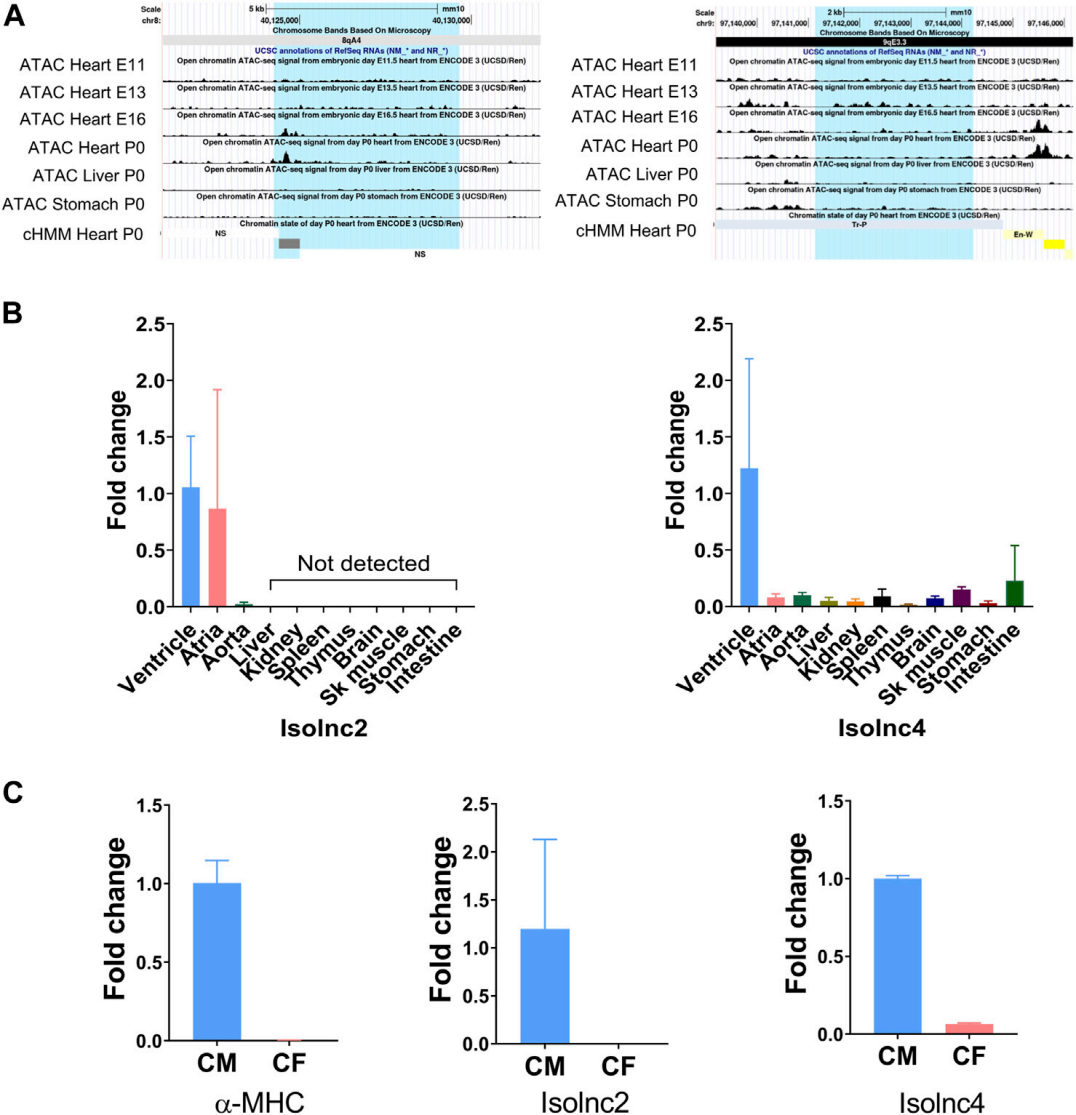
UCSC screenshots of ATAC-seq and qRT-PCR showing expression patterns of Isolncs 2 and 4 in different tissues and subcellular fractions. **(A)** UCSC Genome Browser (mouse mm10) screenshots using “open chromatin track” showing ATAC sequencing across the heart at E11 to E16 and P0 along with the liver and stomach. **(B)** Tissue profiling of Isolncs 2 and 4 in adult mice. **(C)** Expression of the novel Isolncs in a subcellular fraction of the heart (CM vs. CF).

Following our pipeline for the identification of novel lncRNAs, we obtained a final set of 94 unique transcripts, which we classify as “putative novel lncRNAs” (Figure 4A). Following these criteria, we identified four differentially expressed novel transcripts without coding potential based on the CPAT assessment (Figures 4B, C). Henceforth, we refer to these novel lncRNAs as Isolnc1, 2, 3, or 4 (Table 1). Among the four Isolncs detected, Isolncs 1, 2, and 3 were upregulated, while Isolnc4 showed decreased expression after isoproterenol treatment (Table 1). Notably, Isolnc1 is transcribed in an antisense manner from an intronic region of the *Clip4* gene on chromosome 17, while the other three are intergenic (Table 1). Isolnc2 is located approximately 36 Mbp downstream of the protein-coding gene *Fgf20* on chromosome 8, while Isolnc3 positioned between the protein-coding genes *Ndufa6* and *Cyp2d22* on chromosome 15 (Table 1). Another intergenic non-coding transcript Isolnc4 was found 30 kb upstream to *Slc25a36* (Table 1). To validate the results from RNA sequencing, individual qRT-PCR analysis was performed for Isolncs (Supplementary Figure S4B). We prepared primers for these Isolncs and observed no expression in the absence of reverse transcriptase, suggesting no genomic DNA contamination in our cDNA preparation (Supplementary Figure S4A). Similar to RNA sequencing data, the expression of Isolnc2 is upregulated, while Isolnc4 is downregulated upon isoproterenol treatment (Figure 4D). Organ profiling of the Isolncs further indicated heart-specific expression of Isolncs 2 and 4 (Figure 5B; Supplementary Figure S4C). Based on these observations, we selected Isolncs 2 and 4 for further analysis.

**TABLE 1:** (A) Chromosomal coordinates of the different novel transcripts obtained from 4, 8, and 11 day groups.

Condition	Chromosome location	Transcript name	Strand	Type of transcript	Neighboring genes	Expression pattern
4D	Chr17:71779738–71781873	Isolnc1	-	Intronic	*Clip4*	Upregulated
8D & 11D	Chr8:40124250–40129676	Isolnc2	+	Intergenic	*Msr1* and *Fgf20*	Upregulated
8D	Chr15:82361513–82368690	Isolnc3	+	Intergenic	*Ndufa6* and *Cyp2d22*	Upregulated
8D	Chr9:97141142–97144234	Isolnc4	+	Intergenic	*Slc25A36* and *Trim42*	Downregulated

We performed lncRNA–mRNA co-expression analysis on Isolnc2 and Isolnc4. Isolnc2 exhibited detectable expression levels at both 8 and 11 days, and in both instances, its expression displayed a robust positive correlation with certain genes, such as BMP and activin membrane-bound inhibitor (*Bambi*), aryl hydrocarbon receptor nuclear translocator-like protein 1 (*Arntl/Bmal1*), serine proteinase inhibitor A3 (*Serpina3*), and peptidylglycine-α-amidating monooxygenase (*Pam*) (Supplementary Figures S3A, B). BAMBI, known for its negative modulation of TGF-β signaling and cardioprotective effects during pressure overload-induced fibrosis (Villar et al., 2013), was among the positively correlated genes. *Arntl* belongs to the circadian clock-related genes, and mice with CM-specific deletion of *Arntl* displayed increased cardiac remodeling and fibrosis upon TAC or AngII treatment (Young et al., 2014).

Conversely, among the genes displaying negative correlations with the lncRNA expression were resistin-like alpha (*Retnla*), circadian-associated repressor of transcription (*Ciart*), nuclear receptor subfamily 1 group D member 1 (*Nr1d1*), and stearoyl-CoA desaturase (*Scd4*) (Supplementary Figures S3A, B). *Retnla* is a marker for activated anti-inflammatory macrophages, and transgenic *Retnla* mice exhibited worsened cardiac function and cardiomyocyte apoptosis (Kim et al., 2021). *Nr1d1* and *Scd4* have also been implicated previously in cardiac development and injury (Miyazaki et al., 2003; Zhao et al., 2022).

Isolnc4 displayed a strong positive correlation with genes such as Perilipin 5 (*Plin5*) and angiopoietin-like protein 3 (*Angplt3*), both associated with lipid metabolism (Stitziel et al., 2017; Wang et al., 2019; Cinato et al., 2023). However, a notable negative correlation was observed with genes involved in ANP secretion, such as *Pam* (Bäck et al., 2020), and TGF-β regulation, such as *Bambi* (Villar et al., 2013) (Supplementary Figures S3A, B). Further investigations are warranted to establish a direct link between lncRNA expression and these biological processes.

### Tissue-specific expression and sequence conservation of Isolnc2 and Isolnc4

We further looked into these two transcripts for their chromatin structure and conservation status. Active transcription is characterized by an increase in the local accessibility or “openness” of the chromatin structure. Techniques such as DNaseI hypersensitivity and ATAC sequencing are widely employed to measure these conformational changes (Pique-Regi et al., 2011; Grandi et al., 2022). In this study, we utilized the ENCODE database’s ATAC-seq data and examined the “open chromatin track” settings on the UCSC genome browser. We focused on ATAC-seq peaks from embryonic days E11.5 to E16.5 and P0 and investigated the peaks in the heart and other tissues like the liver and stomach. Isolnc2 is specifically expressed in the heart. ATAC-seq peaks were observed at E16.5, coinciding with the completion of heart morphogenesis (Krishnan et al., 2014), and also at P0, indicating its expression gets enhanced as the heart is moving to maturation, while the peaks were absent in the liver and stomach (Figure 5A). The ChromHMM framework predicted this peak region as an enhancer (Figure 5A). For Isolnc4, stronger ATAC-seq peaks were maximally detected at E13.5, and these peaks were also present in the liver and stomach tissues (Figure 5A). According to the ChromHMM framework, the open chromatin at these sites corresponds to enhancer or TSS-like DNA elements. To validate these ATAC-Seq peaks, we further performed tissue profiling in 10 different organs of mice. Isolnc2 was found to be heart- and vasculature-specific with no detectable expression in other tissues, while Isolnc4 was enriched specifically in the ventricles of the heart (Figure 5B).

The heart is a composite structure of different cell types like myocytes, fibroblasts, and endothelial cells (Gray et al., 2018). We checked the expression of Isolnc2 and Isolnc4 in fractionated heart samples, and both were found to be enriched in myocytes (Figure 5C).

Functionally relevant lncRNAs generally exhibit sequence or structural conservation across different species. To investigate this, we utilized the “comparative genomic” settings in the UCSC platform. Isolnc2 exhibited a high degree of conservation among various primate and placental mammal species, while Isolnc4 displayed modest conservation (Supplementary Figure S5A).

## Discussion

Chronic heart failure (CHF) is characterized by significant damage to the myocardial tissue and impaired contractile function (Mudd and Kass, 2008). As the heart matures, the contractile myocytes lose their ability to divide (Zak, 1973). Any injury that triggers the loss of these cells leads to the invasion of non-contractile cells, followed by impairment of cardiac contractility (Zak, 1973; Díez et al., 2005). Several animal models have been described to study pathological changes associated with cardiovascular diseases (Deshaies et al., 1981; Frey et al., 2004; Gao et al., 2010; Houser et al., 2012). In animal models, myocardial disease is generally induced by surgery (Houser et al., 2012; Richards et al., 2019), genetic manipulation (Chu et al., 2002; Tobin et al., 2017), or pharmacological treatment (Gavras et al., 1971; Benjamin et al., 1989; Golomb et al., 1994; Rocha et al., 2002; Feng and Li, 2010). Despite some limitations, these models offer valuable insights into the intricacies of the disease (Frey et al., 2004; Feng and Li, 2010).

Surgical models such as TAC or CAL closely mimic pathological changes that are associated with cardiac hypertrophy or myocardial infarction (Golomb et al., 1994; Gao et al., 2010; Houser et al., 2012; Richards et al., 2019). However, these models are labor-intensive, and often present challenges regarding their success rate and high mortality. Alternatively, genetic approaches like transgenic overexpression or gene-knockout models (Tobin et al., 2017), as well as drug-based approaches like angiotensin II or isoproterenol infusion, have been extensively employed (Deshaies et al., 1981; Taylor and Tang, 1984; Golomb et al., 1994).

Isoproterenol is a well-studied β-adrenergic agonist that activates the β1 adrenergic receptors in the heart, leading to cardiac hypertrophy, which progresses gradually toward fibrosis and heart failure (Park et al., 2018; He et al., 2019). A moderate dose of isoproterenol (4–60 mg/kg/day) can induce cardiac hypertrophy and fibrosis (Oudit et al., 2003; Park et al., 2018; Grant et al., 2020; Pan et al., 2022) in 7–15 days; the TAC model takes approximately 4–8 weeks to develop fibrosis (Li et al., 2022). Although TAC is an excellent model that very closely mimics pathological conditions like aortic stenosis, isoproterenol serves as an alternative and quick method to develop fibrosis. Previous studies have compared the transcriptome of mice that underwent TAC for 2, 4, and 8 weeks (Li et al., 2022). There are several similarities in cardiac remodeling induced by TAC vs. induced by isoproterenol. For example, decreased expression of the genes involved in fatty acid metabolic processes and cardiac contraction starts from an early timepoint such as 2 weeks post-TAC and 4 days post-isoproterenol administration (Li et al., 2022). In isoproterenol-induced cardiac remodeling, ECM gene deregulation was observed as early as 4 days (Figure 1E). However, Li et al. (2022) showed that deregulation in the ECM gene expression starts only after 4 weeks post-TAC, indicating isoproterenol-based models induced cardiac remodeling and fibrosis at the early stage and could be a preferred model for studying fibrosis.

The extracellular matrix (ECM) is a non-contractile component of the heart that is secreted by cardiac fibroblasts and plays a crucial role in maintaining the heart’s structure and function (Díez et al., 2005). Any form of cardiac injury eventually leads to cardiac remodeling, disrupting the delicate balance between ECM formation and degradation. Initially, this remodeling process acts as a compensatory mechanism, but with sustained stress, it drives the heart toward failure (Porter and Turner, 2009). Increased ECM deposition can result in cardiac dysfunction and arrhythmias (Porter and Turner, 2009). In our study, picrosirius staining on horizontal cross sections of the isoproterenol-treated hearts revealed the onset of cardiac fibrosis from day 4 after the treatment.

Transcriptome analysis using RNA sequencing helps in comprehensive understanding of the changes occurring during cardiac hypertrophy and heart failure. In the present study, transcriptome profiling identified four distinct gene clusters to be deregulated during isoproterenol-induced cardiac fibrosis. All the deregulated genes are shown in Supplementary Tables S4, S5. The genes that are primarily associated with ECM deposition, cell adhesion, and wound healing processes were significantly affected starting from day 4 after isoproterenol treatment, while RNA splicing and processing genes are affected from day 8 onward.

Based on our analysis utilizing Gene Ontology and maSigPro-based time-course analysis, we have observed that the group subjected to 4-day treatment exhibited the most substantial increase in the expression of genes related to ECM remodeling and all structural genes. Notably, well-established ECM remodeling markers such as periostin, collagens, and MMPs are notably elevated, indicating that the remodeling process commences as early as 4 days, following isoproterenol treatment, which is in line with previous studies (Dewenter et al., 2022). Furthermore, the expression of ECM-related genes begins to decline in tandem with a reduction in fatty acid oxidation and aerobic respiration upon prolonged treatment. Interestingly, there is no significant disparity in ECM gene transcription between the saline group and the group treated for 11 days. However, the 11-day treatment group, which represents the later stages of heart failure, does exhibit diminished expression of genes associated with cardiac contraction and RNA processing.

In both prenatal and adult mammalian hearts, fatty acid and lipid metabolism are well-known to play a crucial role in maintaining the pump efficiency (Marin-Garcia and Goldenthal, 2002; Lopaschuk et al., 2021). Failing hearts undergo significant alterations in cardiac metabolism and respiration, shifting their fuel preference from fatty acid metabolism to glucose, following injury (Marin-Garcia and Goldenthal, 2002). These changes have profound effects on the cardiac structure and overall function. Interestingly, we observed a similar decrease in fatty acid metabolism with prolonged isoproterenol treatment (Supplementary Figure S2).

Puromycin incorporation assay indicates protein synthesis. Using this method, it was shown that protein synthesis significantly increases after day 3 of isoproterenol induction, followed by a sharp decline (Ravi et al., 2018). Consistent with their findings, our transcriptome data also revealed a steady rise in genes encoding ribosomal proteins at day 4 of isoproterenol treatment, followed by a decline at days 8 and 11 (Supplementary Figure S2).

Less than 2% of the total human genome codes for proteins. Recent evidence suggests that non-coding RNAs play a crucial role in several physiological and pathological processes. Importantly, miRNAs and lncRNAs have emerged as critical players in several disease mechanisms. In the present study, we identified several well-characterized lncRNAs that exhibited altered expression during isoproterenol-induced fibrosis. Notably, we also found several novel transcripts without coding potential to be deregulated upon isoproterenol treatment. We refer to these novel lncRNAs as Isolnc 1, 2, 3, or 4. Among these, Isolnc2 and Isolnc4 are exclusively expressed in the heart, while Isolnc2 is upregulated during isoproterenol-induced cardiac fibrosis and Isolnc4 is downregulated. Further analysis revealed that these two lncRNAs formed several co-expression gene pairs with DEGs (Supplementary Figures S3A, B; Supplementary Tables S7, S8). The co-expressing pairs revealed several genes which have been well-studied in several heart diseases. Further characterization and experimental validation of these Isolncs will give a better understanding regarding these associations.

This study gives a broader insight into the transcriptome landscape of isoproterenol-induced fibrosis. We identified four novel lncRNAs, out of which we could validate two lncRNAs using qRT PCR, and found them to be heart enriched. Further investigation of these novel Isolncs may give more insights on the role of these candidates in cardiac fibrosis.

## Data Availability

The datasets presented in this study is available in GEO database with accession GSE239653.
