# Supplementary material for: Transcriptional changes during isoproterenol-induced cardiac fibrosis in mice
Source: Front Mol Biosci. 2023 Dec 18;10:1263913. doi: 10.3389/fmolb.2023.1263913 (PMC10765171; doi:10.3389/fmolb.2023.1263913)
Supplement: Supplementary file 4 [file DataSheet1.PDF]

(A)

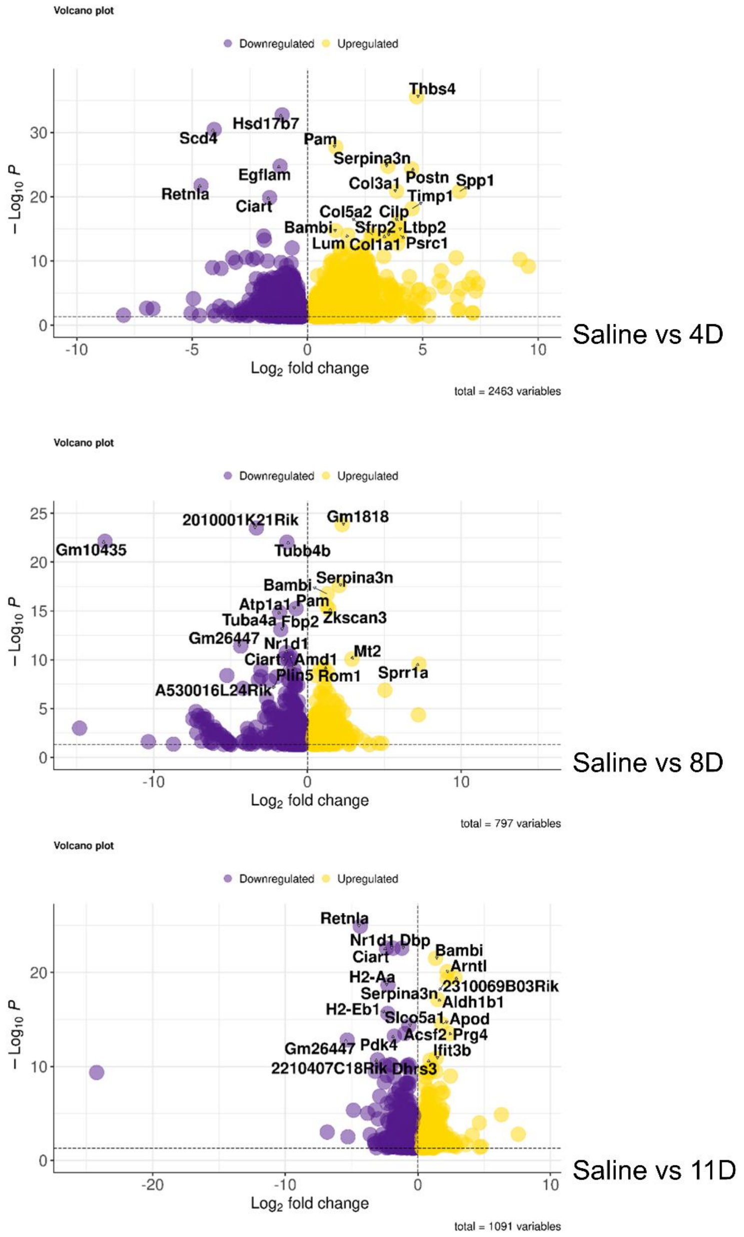

Supplementary Figure 1

Cluster 2

Respiration

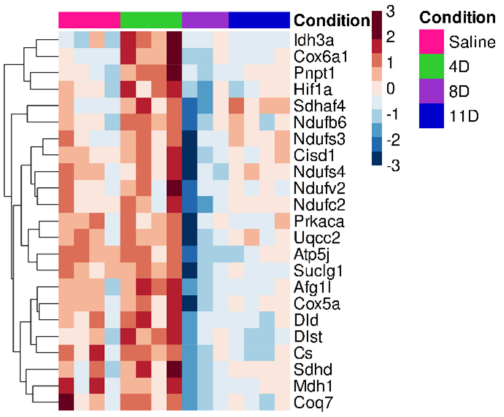

Ribosome Biogenesis

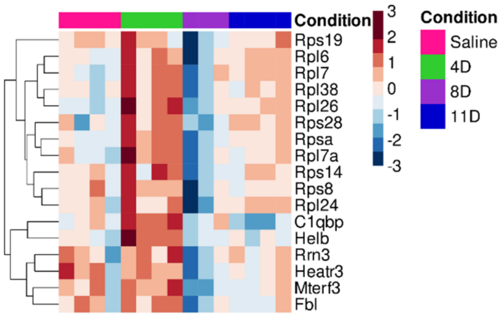

Cluster 3

Fatty Acid Metabolism

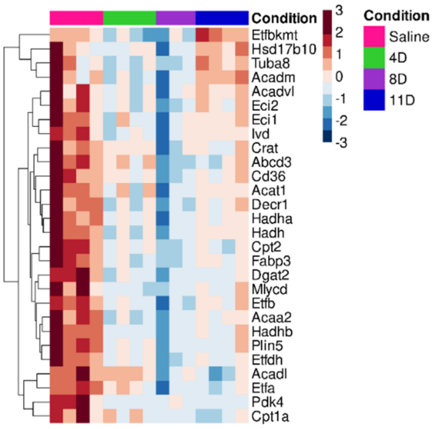

Supplementary Figure 2

(A)

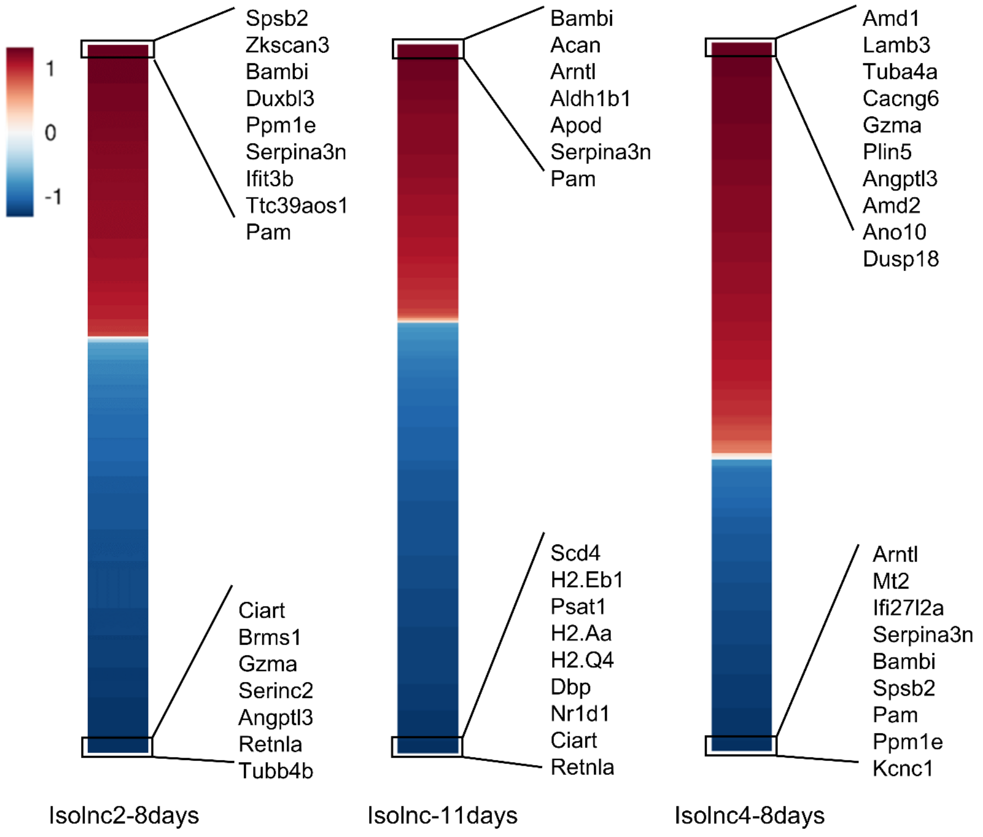

(B)

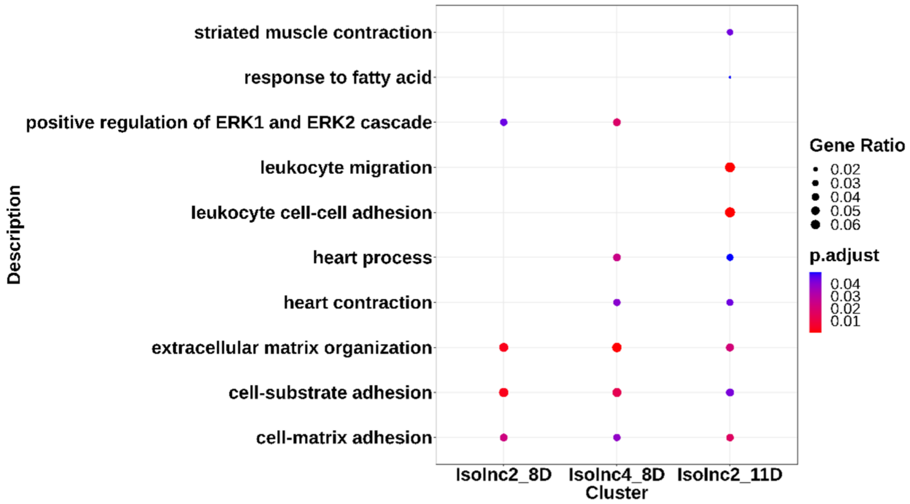

Supplementary Figure 3

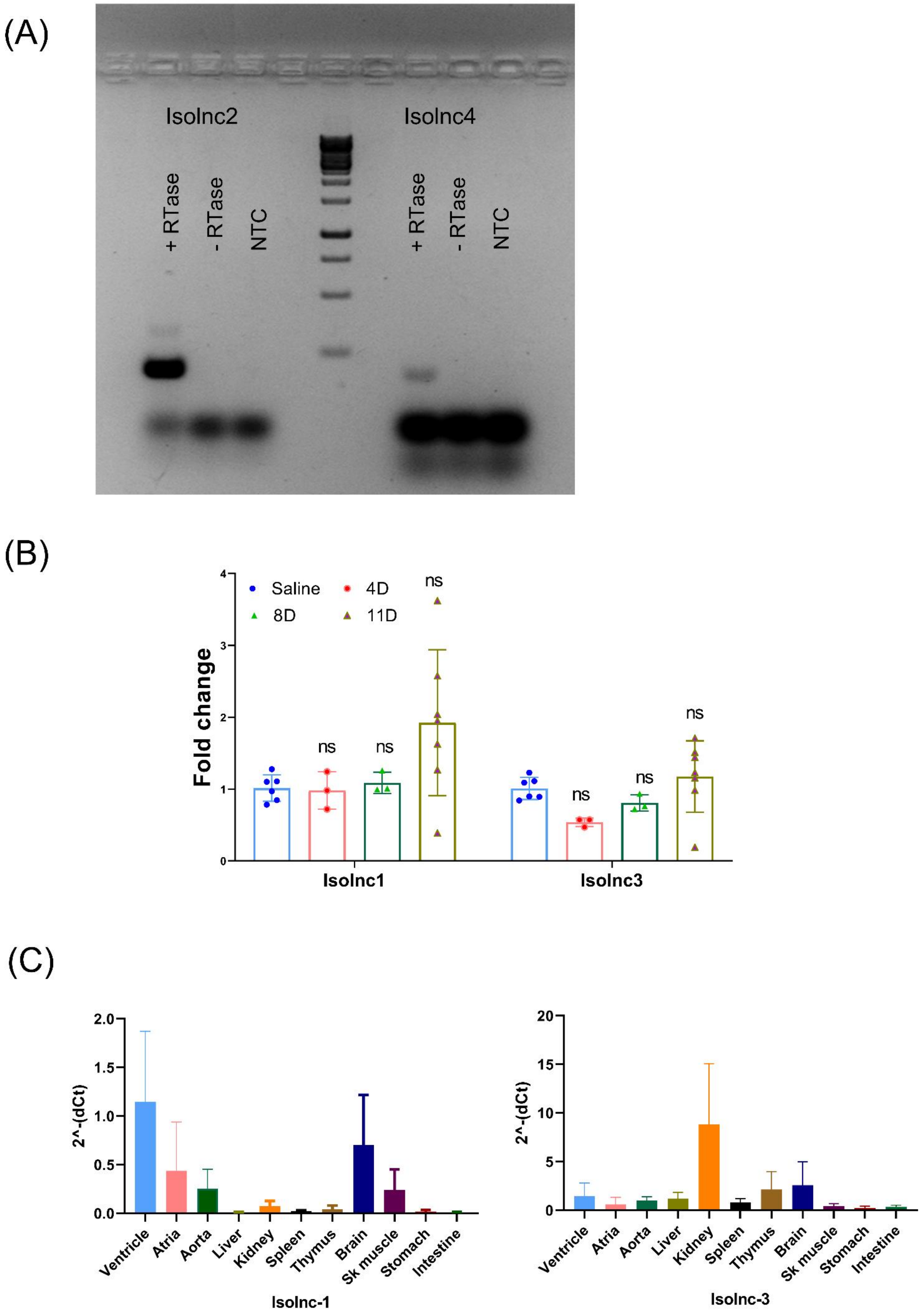

Supplementary Figure 4

(A)

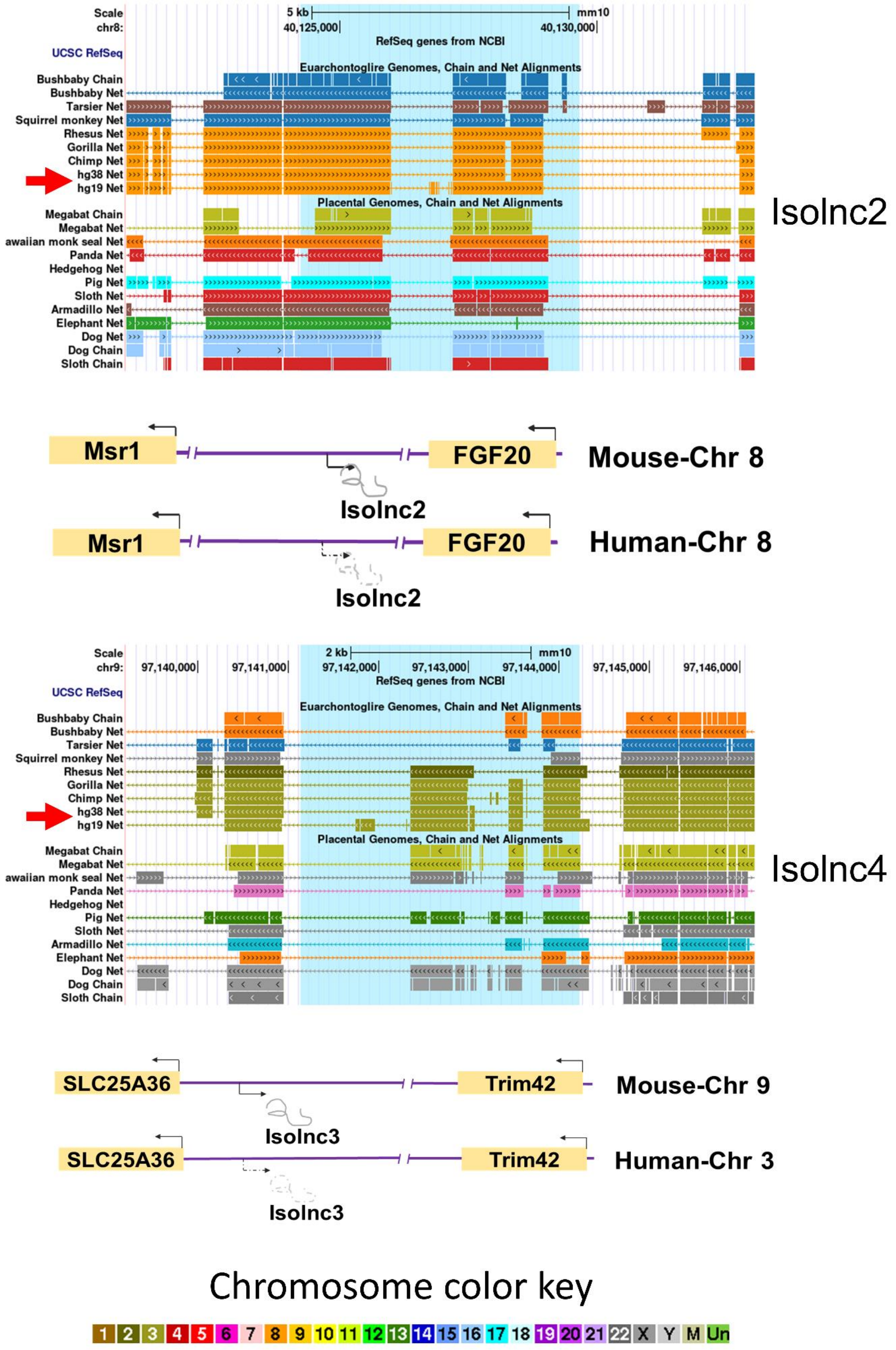

Supplementary Figure 5

S1: (A) Volcano plots showing the DEGs in 4,8 and 11 days isoproterenol treated group.

S2: Heatmap depicting the gene expression pattern of some pathways obtained from each clusters.

S3: (A) Heatmap of the Isolncs against its coexpressing gene pairs. (B) Gene ontology showing the different possible pathways affected by the co-expressing gene pairs (Isolnc2 and Isolnc4 vs DEGs of the respective groups).

S4: Primer validation of all novel transcripts (A) PCR showing the expression of novel LncRNAs in the presence and absence of Reverse Transcriptase (B) qRT PCR of Isolnc1 And 3. (c) Organ profiling of Isolnc1 and 3. Oneway ANOVA using Dunnett's test was performed to compute statistical differences between saline and isoproterenol groups.

S5: (A) Screenshots using 'comparative genomics' settings in UCSC show conservation status among different mammals.
